# Risk Factors for Chronic Pain in Women: The Role of Violence Exposure in a Case–Control Study

**DOI:** 10.3390/life15060976

**Published:** 2025-06-18

**Authors:** Allison Uvelli, Erica Pugliese, Fabio Ferretti

**Affiliations:** 1 Department of Medical Science, Surgery, and Neurosciences, University of Siena, 53100 Siena, Italy; ferrefa@unisi.it; 2 Department of Clinical Psychology, University of Amsterdam, 1012 WS Amsterdam, The Netherlands; e.pugliese@uva.nl

**Keywords:** gender violence, chronic pain, risk factors, case–control study, odds ratio

## Abstract

Background: Chronic pain is an unpleasant sensory and emotional experience that greatly affects functioning and well-being. Studies link chronic pain and violence against women, with an odds ratio of 2.08 and a 26% prevalence rate. The bio-psycho-social consequences reduce quality of life and cause disability. Despite extensive research, the etiology remains unclear. This study investigates the bio-psycho-social risk factors of chronic pain in women, both victims and non-victims of violence. Methods: A case–control study (December 2023–June 2024) used odds ratios and Fisher’s exact test to explore risk factors associated with chronic pain. Univariate logistic regressions identified significant predictors. Results: The study included 170 women (68 victims), half with chronic pain. Nine risk factors were specific to victims (three biological, six psycho-social), four to non-victims (two biological, two psycho-social), and twenty-three to all women (five biological, eighteen psycho-social). A four-factor model best explained risk in victims and all women, while a two-factor model fit non-victims. Conclusions: The bio-psycho-social model of chronic pain is supported, identifying specific risk factors. These findings can aid anti-violence and healthcare professionals in screening and early intervention.

## 1. Introduction

Chronic pain is defined as persistent pain lasting at least three months that significantly affects an individual’s functioning or overall well-being [1]. The International Association for the Study of Pain (IASP) characterizes it as “an unpleasant sensory and emotional experience associated with, or resembling that associated with, actual or potential tissue damage” [2]. Numerous studies have explored various types of chronic pain, such as pelvic or vaginal pain [3], fibromyalgia [4], irritable bowel syndrome and other gastrointestinal symptoms [5], abdominal pain [6], migraine or headache [7], and back and neck pain [8,9], all of which frequently become chronic conditions.

These conditions are more prevalent among women than men, both globally and within specific regions. Globally, an estimated 30% of the adult population experiences chronic pain, with women consistently showing higher prevalence rates than men [10]. In Europe, the Survey of Chronic Pain reports that 21% of adults are affected, with women accounting for nearly 60% of cases [11]. In Italy, approximately 26% of women experience chronic pain compared to 19% of men, according to a report from the Istituto Superiore di Sanità [12]. Similarly, data from the Italian Society of General Medicine (SIMG) estimate that around 27% of Italian women suffer from moderate to severe chronic pain, with prevalence increasing with age [13].

The consequences of chronic pain for women are often profound and multifaceted, affecting physical, psychological, and social well-being. Physically, chronic pain can lead to reduced mobility, fatigue, and difficulties in performing everyday tasks, ultimately diminishing physical function [14]. Psychologically, women with chronic pain are more likely to experience depression, anxiety, and emotional distress, often reporting feelings of helplessness and sleep disturbances that further exacerbate mental health concerns [15,16]. Socially, chronic pain may result in isolation, strained interpersonal relationships, and difficulties maintaining employment, all of which can contribute to financial stress and decreased social participation [11,17]. Taken together, these outcomes significantly lower the quality of life among women with chronic pain.

A major factor consistently linked to the development of chronic pain in women is a history of violence and trauma [18,19,20,21,22]. For instance, studies report that between 48% [23] and 84% [24] of women who have experienced abuse also report chronic pain, with an estimated odds ratio of 2.08. These findings are based on research conducted in diverse regions, not limited to Europe [25]. However, the exact mechanisms underlying the onset of chronic pain in this context are still not fully understood, despite a growing body of evidence supporting this association. One widely accepted hypothesis is that pain may involve psychological components that are not objectively observable, referred to as nociplastic pain [26]. This type of pain is believed to result from altered functioning of the sensory nervous system at both the peripheral and central levels, leading to increased pain sensitivity [27]. Supporting this hypothesis are findings that the location of reported pain often does not correspond to areas of physical injury [28], alongside the lack of specific clinical markers, the subjectivity of the symptoms, and the challenges individuals face in accurately describing their pain experiences [29].

The bio-psycho-social model, widely used in health and trauma research [30], offers a comprehensive framework for understanding how psychological and social stressors, such as interpersonal violence, can influence physical health outcomes like chronic pain. According to this model, illness results from the interplay between biological, psychological, and social factors, which may differ from person to person. As Uvelli and colleagues note [31], identifying such contributing factors can have important clinical and legal implications. Clinically, it may support the development of screening tools aimed at preventing chronic pain and guiding individuals toward appropriate treatment. Legally, it may allow victims to seek compensation for chronic pain arising from experiences of violence.

### Study Objectives

Given the limited understanding of the mechanism underlying chronic pain and the lack of validated tools to assess risk factors, particularly in populations exposed to violence, this study aimed to identify specific risk factors associated with chronic pain in women, with a particular focus on experiences of victimization. This research was conducted in Italy, a country where gender-based violence continues to represent a pressing public health concern. According to the Italian National Institute of Statistics (ISTAT), approximately 31.5% of women aged 16 to 70 have experienced physical or sexual violence in their lifetime [32]. Despite this high prevalence, empirical studies investigating the long-term health consequences of such violence, especially chronic pain, remain limited within the Italian context. A recent systematic review and meta-analysis by Uvelli et al. [25] confirmed the association between violence against women and chronic pain but also underscored a lack of region-specific evidence, particularly from Italy. This gap is critical given the country’s unique cultural, legal, and healthcare characteristics, which may influence both exposure to violence and access to appropriate care. Therefore, the primary objective of this study was to identify and quantify the biological, psychological, and social risk factors associated with chronic pain in women, including but not limited to those related to a history of interpersonal violence. By doing so, the study aimed to contribute locally grounded evidence that can inform screening practices, clinical interventions, and prevention policies tailored to the Italian context.

## 2. Methods

### 2.1. Theoretical Framework

This study was based on the bio-psycho-social model which conceptualizes health outcomes as the result of the interaction between biological, psychological, and social factors. Chronic pain among victims of violence cannot be attributed to a single cause, whether psychological, sociological, or biological, but rather emerges from the dynamic interplay of all three domains. A recent scoping review [31] aimed to identify the key risk factors associated with the development of chronic pain in victims of violence, further highlighting the relevance of this comprehensive framework. The review identified both biological and psycho-social variables grouped into fourteen categories: nine biological and five psycho-social. The biological categories include issues related to weight, acute respiratory infections (upper and lower), genitourinary conditions, cardiovascular symptoms and diseases, endocrine disorders, hormonal imbalances, gastrointestinal problems, skin conditions, and specific inflammations. The psycho-social categories encompass mental health disorders, substance use, significant life events, quality of life, and personal characteristics. Across these fourteen categories, a total of 65 distinct indicators, symptoms, and conditions were identified. However, the precise way in which these factors interact and which among them play a pivotal role in the onset of chronic pain remains unclear. This model served as the conceptual framework for analyzing chronic pain concerning trauma symptoms and exposure to interpersonal violence.

### 2.2. Procedure

This case–control study was conducted between December 2023 and June 2024 and was designed to compare individuals with chronic pain (cases) to those without pain (controls). Data were collected through Italian anti-violence centers, the general population, and chronic pain clinics. All forms of victimization were included, encompassing physical, sexual, and psychological abuse. Chronic pain was defined as “pain lasting for at least three months and negatively affecting the individual’s functioning or well-being” [1], and included conditions such as pelvic/vaginal pain, fibromyalgia, abdominal pain, migraine/headache, back pain, and neck pain. The study was part of a broader research project [33], ultimately aimed at developing a validated assessment tool for forensic evaluations of victims with chronic pain. This tool is intended to distinguish among the biological, psychological, and social components of pain and to support professionals in guiding women toward individualized treatments based on assessment outcomes. In this initial phase, the goal was to identify the bio-psycho-social risk factors associated with chronic pain in victims of violence. The authors affirm that all procedures conducted in this study adhered to the ethical standards of the relevant national and institutional research committees and complied with the 1975 Helsinki Declaration, as revised in 2013. All procedures involving human participants were approved by the Comitato per la Ricerca Etica nelle scienze Umane e Sociali (CAREUS; registration number 31/2023 issued on 6 December 2023) at the University of Siena. All participants provided written informed consent after receiving full information about the study and its objectives.

### 2.3. Participants

Two groups of women, one with chronic pain (cases) and the other without (controls), were selected. The two groups were matched based on age and education level. Participants were recruited through multiple channels to ensure a diverse sample. Women accessing several anti-violence centers were enrolled with the support of the center operators who were informed about the project by the research team. Additionally, women from pain therapy departments were recruited through healthcare professionals involved in their care who were also briefed on the study. Finally, other participants from the general population were recruited via posters displayed in public spaces, which provided detailed information about the project and contact details for interested individuals. Data were collected by administering the checklist face-to-face or online.

### 2.4. Inclusion and Exclusion Criteria

- Inclusion criteria: female participants aged 18 years or older, able to understand and speak Italian. Participants could have a diagnosis of chronic pain or not and could have experienced at least one instance of victimization (physical, sexual, and/or psychological) or not.
- Exclusion criteria: male participants, individuals under 18 years of age, those not fluent in Italian, as well as individuals with severe mental health conditions that prevented informed participation or who were unable to provide valid informed consent.

### 2.5. Measure

Based on the scoping review by Uvelli et al. [31], a specific checklist was created and administered to assess risk factors of chronic pain. It was composite by two macro-areas, biological and psycho-social factors, subdivided into 14 categories: weight conditions, acute upper/lower respiratory tract infection, genitourinary conditions, cardiovascular symptoms and conditions, endocrine disease, hormonal conditions, gastrointestinal disorders, skin problems, specific inflammations, mental health disease, use of psychoactive substances, life events (including victimization experiences), life quality, and personal characteristics. The checklist comprised 72 items with a binary response (yes/no) that evaluates the presence or absence of each condition. The checklist used to assess risk factors is currently undergoing validation; a complete copy is available in Supplementary Materials (please refer to Table S1 in Supplementary Materials for more information).

### 2.6. Statistical Analysis

The sample size was calculated according to the quality of OR estimation: considering a relative precision of 0.5, a level of confidence of 95%, an expected prevalence of the outcome in the absence group of 0.2, and an expected OR of 7, the sample size was determined as 170 women (85 in each group). Age and education level, the only individual characteristics collected, were summarized using mean and standard deviation. The odds ratio and its 95% confidence interval explored the existence of risk factors. In contrast, the non-parametric Fisher’s exact test analyzed the association between exposure to bio-psycho-social factors and the related outcome (chronic pain). Each estimation was executed in the victim’s group, the non-victim group, and the whole sample of women enrolled in the study. A binary univariate logistic model was used to confirm the contribution of each risk factor to chronic pain. Multivariate binary logistic regressions were fitted to victims and non-victims, and the total sample was analyzed to determine the relevance of the significant risk factors in predicting chronic pain. Given the exploratory nature of this study and the large number of variables analyzed, formal corrections for multiple testing (e.g., Bonferroni, FDR) were not applied to avoid inflating Type II errors. To address potential false positives, any *p* value ranging from 0.02 to 0.05 was considered nominally significant, and only variables showing significance in univariate analyses were included in subsequent multivariate logistic regression models, which helped identify the most robust risk factors. The significance level was set at *p* < 0.05, and data analysis was executed with Jamovi (v 2.3.0.0).

## 3. Results

A total of 85 cases (chronic pain) and 85 controls (pain-free) were enrolled, of which 68 were victims of violence (42 cases and 26 controls). Both groups had a mean age of 43.6 (SD = 12.7) and years of education of 15.8 (SD = 3.5).

### 3.1. Risk Factors of Chronic Pain in Victims

The “transmissible sexual infections” risk factor was not analyzed as no women had this condition. Within social risk factors, certain conditions such as having good relationships with parents, feeling satisfied with one’s life as it is, having high self-esteem, experiencing sexual satisfaction and desire, maintaining meaningful friendships, receiving social support, and engaging in physical activity or sports, behave like reverse items and can act as protective factors. In fact, the lack of these conditions is more likely to serve as a risk factor for the development of chronic pain. Of the 72 analyzed risk factors, only 9 were distinctive to the victims. Specifically, within the biological category, respiratory infection in victims increased the risk of the onset of chronic pain by 4.72 (*p* = 0.02), respiratory allergies had an OR of 3.83 (*p* = 0.05), and lipid metabolism disorder had that of 8.87 (*p* = 0.02). Regarding the psycho-social conditions, victims with an anxiety disorder had an increased risk of developing chronic pain of 4.36 (*p* = 0.009), those without social support had an OR of 5.37 (*p* = 0.03), and those who had more than four sexual partners during life had an OR of 2.92 (*p* = 0.04) to develop chronic pain. Lastly, concerning the abuse conditions, only when they occur at an adult age do they represent significant risk factors for chronic pain. This correlation is particularly strong/frequent in the case of a single sexual abuse (OR: 3.74, *p* = 0.03), recurrent physical abuse (OR: 4.26, *p* = 0.04), and recurrent psychological abuse (OR: 4.40, *p* = 0.01) by the partner. Two abuse conditions reached significance only when the sample size increased, i.e., single physical abuse (OR: 3.15, *p* = 0.04), and recurrent psychological abuse during childhood (OR: 3.16, *p* = 0.01).

### 3.2. Risk Factors of Chronic Pain in Non-Victims

The conditions of muscle inflammations (OR: 18.63, *p* ≤ 0.001), osteoarthritis (OR: 6.51, *p* = 0.01), having any psychological disorder (OR: 7.54, *p* = 0.008), and satisfaction for one’s life as it is (OR: 2.82, *p* = 0.02), are the only specifically preeminent in non-victims.

### 3.3. Risk Factors of Chronic Pain in Women

Considering the total sample, there are many more risk factors than those only present in victims. Of biological condition, urinary infections predispose all women to chronic pain (OR: 6.64, *p* ≤ 0.001), such as vaginal swelling (OR: 16.34, *p* = 0.01), heart palpitations (OR: 3.91, *p* ≤ 0.001), alterations of intestinal transit (OR: 2.64, *p* = 0.03), and skin problems/irritation (OR: 3.22, *p* = 0.002). Of psycho-social conditions, results show the high impact of psychological diseases, such as sleep (OR: 4.10, *p* ≤ 0.001), mood (OR: 5.36, *p* ≤ 0.001), and somatic (OR: 2.46, *p* = 0.04) disorder, somatizations in general (OR: 7.18, *p* ≤ 0.001), and PTSD (OR: 5.10, *p* ≤ 0.001). Traumatic areas during both childhood (OR: 2.10, *p* = 0.04) and adulthood (OR: 2.04, *p* = 0.03) impact pain, regardless of developing a full-blown disorder, and even levels of stress (OR: 2.34, *p* = 0.01). Having tense relationships with both current (OR: 2.36, *p* = 0.01) and origin family (OR: 2.15, *p* = 0.02) and not having a good relationship with parents (3.89, *p* = 0.004) also represent a risk. Finally, transdiagnostic features such as suicidal thoughts (OR: 6.34, *p* = 0.003), guilt and shame (OR: 2.37, *p* = 0.01), and high emotionality (OR: 2.13, *p* = 0.03) have a link with chronic pain, as does the tiredness/absence of energy (OR: 5.77, *p* ≤ 0.001) and the impediment in normal activity due to acute pain (OR: 12.3, *p* ≤ 0.001) (for a complete overview of the results, please refer to Table S2 in Supplementary Materials).

### 3.4. Summary of the Risk Factor Evidence

At this point, we can see only significant risk factors in descending hierarchical order (Table 1).

### 3.5. Binary Logistic Regression Results

The assumptions of logistic regression were tested using only the risk factors significantly related to the victims and the 68-victim sample to determine which had a greater impact on causing the symptoms. Chronic pain was the dependent variable, and a four-factor model explains 68% of the total statistical deviance. The most influential categories are lipid metabolism disorder, respiratory allergies, recurrent psychological abuse at adult age, and single sexual abuse at adult age (Table 2). Of the 102 non-victims, the 2 most influential categories are muscle inflammations and any psychological disorders (Table 3). Lastly, considering the entire sample and the risk factors significant for all women, a different four-factor model shows that urinary infections, impediments in daily activities, tiredness/absence of energy, and sleep disorders are the most crucial for the onset of chronic pain (Table 4).

## 4. Discussion

Violence, including physical, sexual, and psychological abuse, is a major risk factor for negative mental and physical health outcomes. While the mental health effects of abuse are well documented [23,34] and its long-term physical impacts are increasingly recognized [18,35], far less is known about how violence contributes to chronic pain [25,33]. This study, using a bio-psycho-social model [30], systematically explores the biological, psychological, and social risk factors for chronic pain development, a severely debilitating condition that often goes unrecognized both by healthcare professionals and by those who suffer from it due to violence. By addressing this gap, the study offers a detailed scientific comparison of the bio-psycho-social risk factors for chronic pain among victims, non-victims, and women in general. This study identifies nine risk factors specific to victims (three biological and six psychosocial), four specific to non-victims (two biological and two psychosocial), and twenty-three applicable to women in general (five biological and eighteen psychosocial).

### 4.1. Biological Risk Factors for Chronic Pain Among Victims

Victims of violence showed a higher prevalence of lipid metabolism disorders, respiratory allergies, and recurrent respiratory infections, patterns that may reflect the physiological burden of chronic stress. Rather than viewing these conditions as isolated comorbidities, they can be understood as interconnected outcomes of stress-related dysregulation in metabolic and immune systems.

The link between lipid metabolism disorders and victimization may be explained by the persistent activation of the hypothalamic–pituitary–adrenal (HPA) axis, which affects metabolic processes and increases vulnerability to obesity and cardiovascular disease, both associated with chronic pain conditions [36,37,38,39]. Similarly, the elevated rate of respiratory allergies could signal heightened immune reactivity, as chronic stress promotes systemic inflammation and hypersensitivity responses [40,41,42].

Recurrent respiratory infections among victims may indicate a suppressed immune function, another consequence of prolonged stress exposure. Elevated cortisol levels, commonly found in victims of violence, can reduce the body’s ability to fight infections, leading to a cycle of repeated illness and inflammation that sustains or worsens chronic pain over time [36,40,43,44].

These findings support an integrative view of chronic pain in victims, not merely as a somatic issue but as the expression of systemic physiological dysregulation rooted in trauma. They suggest that clinical assessment and intervention should routinely include metabolic and immunological screening to identify at-risk individuals and guide interdisciplinary care.

### 4.2. Psycho-Social Risk Factors for Chronic Pain Among Victims

Among the psycho-social risk factors for chronic pain, the absence of social support emerges as the most significant, followed by anxiety disorders and a higher number of lifetime sexual partners. These factors point to a complex interplay between emotional regulation, interpersonal stress, and somatic symptoms in victims of violence.

Lack of social support appears to exacerbate the experience of chronic pain by undermining emotional resilience and amplifying stress-related responses. Social relationships serve as buffers against adversity, and their absence is consistently linked to greater pain sensitivity and poorer health outcomes in trauma survivors [45,46]. In victims of violence, isolation may further compound the biological stress burden, reinforcing pain pathways over time.

Anxiety disorders represent another important risk factor, not only due to their emotional toll but also because of their physiological impact. Anxiety can heighten attention to bodily sensations, amplify pain perception, and contribute to a state of persistent hyperarousal [47]. These psychological mechanisms are often reinforced by alterations in the HPA axis and inflammatory responses associated with chronic anxiety [48]. Among violence-exposed individuals, anxiety is particularly prevalent and often accompanied by hypervigilance and muscle tension, which may contribute to somatic symptoms such as myalgia and inflammation, increasing the likelihood of chronic pain [49,50,51].

The association between a higher number of sexual partners and chronic pain may indicate relational instability or accumulated relational stressors. Frequent changes in intimate relationships can expose individuals to attachment disruptions and emotional distress, contributing to cumulative stress and consequently heightened pain vulnerability [52]. Moreover, relational instability is associated with increased rates of anxiety and depression [53], both of which are well-documented contributors to chronic pain [54]. It is important to interpret this finding within a sociocultural and trauma-informed framework, acknowledging that a higher number of sexual partners may reflect a variety of normative or contextual factors and should not be pathologized. These findings highlight the need for trauma-informed interventions that address not only individual psychopathology but also social and relational dimensions of victims’ lives. Chronic pain in this context may be understood as an embodied expression of psychological and interpersonal wounds that remain unhealed.

### 4.3. The Role of Different Types of Abuse in Adulthood as a Risk Factor for Chronic Pain

In a recent systematic review of 37 studies, Uvelli et al. [25] analyzed research articles focused on women with and without a history of sexual, physical, and emotional abuse. The findings revealed strong associations between violence and chronic pain, indicating that women who experienced violence in adulthood are twice as likely to develop chronic pain conditions.

In line with that, the present study examined specific abuse conditions to determine which types are significantly associated with chronic pain risk. Results showed that only abuse occurring in adulthood emerges as a robust predictor of chronic pain. This correlation is particularly pronounced for recurrent psychological abuse, followed by recurrent physical abuse, and lastly single instances in sexual abuse by a partner. Notably, two additional abuse conditions, recurrent psychological abuse during childhood and single incidents of physical abuse, show statistical significance only when analyzed within larger sample sizes.

These findings suggest that abuse experienced in adulthood may have a more direct and immediate impact on the risk of developing chronic pain compared to abuse during childhood the effects of which may be more diffuse or mediated over time. Studies show that adults exposed to recurrent psychological or physical abuse tend to have higher levels of stress-related inflammation, contributing to chronic pain conditions [55]. Repeated psychological abuse in particular can lead to major pain sensitization, where the nervous system becomes more reactive to pain stimuli, making even minor physical stressors potentially painful [56]. Recurrent physical abuse has a similar impact by causing repeated physical trauma, which can result in lasting damage to musculoskeletal structures, inflammation, and heightened pain sensitivity [27].

Single instances of sexual abuse, although not repeated, are highly traumatic events that can increase vulnerability to chronic pain through altered stress response pathways [43]. Interestingly, abuse occurring during childhood, such as recurrent psychological abuse, shows weaker statistical effects unless sample sizes are large, possibly due to its long-term developmental mediation and interaction with protective factors [57]. For example, one of these factors could be resilience, a well-known buffer for the effect of continuous stress or early adverse experiences on adult functioning [58,59,60].

Additionally, while single incidents of physical abuse show a weaker direct association with chronic pain, they may still contribute to a cumulative risk when combined with other adverse life experiences [47]. This pattern underscores the importance of considering both the type and timing of abuse when evaluating chronic pain vulnerability among survivors.

Psychological abuse in adulthood appears to pose a higher risk for chronic pain than physical or sexual abuse, possibly due to its prolonged, subtle nature that induces continuous stress without visible injuries. Unlike physical abuse, which may have clear endpoints, psychological abuse can be pervasive and insidious, leading to sustained stress responses. This prolonged stress is linked to “hyperarousal,” a heightened reactivity in the nervous system that can contribute to central sensitization, where the body overreacts to pain signals [61].

Sexual abuse is also a significant risk factor, correlating with various health issues, including chronic pain, yet often through mechanisms like acute trauma responses and direct bodily harm [62]. In contrast, psychological abuse fosters a cycle of hypervigilance and helplessness, amplifying both physical and emotional pain [55].

In summary, while both psychological and sexual abuse are critical risk factors, the chronic and cumulative impact of psychological abuse on the nervous system may explain its stronger association with chronic pain. These findings highlight the need for comprehensive clinical attention to psychological abuse, not only for its mental health effects but also for its long-term physical consequences. This also emphasizes the need for tailored interventions targeting each abuse type’s specific effects [63].

### 4.4. An Integrated Perspective on Chronic Pain Risk Among Victims

Taken together, these findings suggest that chronic pain among victims of violence is not attributable to isolated biological or psychosocial factors but rather to the cumulative effects of chronic stress, trauma, and social vulnerability. The high prevalence of lipid metabolism disorders, respiratory allergies, and frequent infections may reflect the long-term physiological toll of sustained HPA axis activation and immune dysregulation, a typical outcome of chronic stress exposure in trauma survivors. In parallel, psychosocial risk factors such as lack of social support and anxiety disorders point to a breakdown in the social buffering systems that normally mitigate stress responses.

Furthermore, the prominent role of psychological abuse, more than physical or sexual abuse, in predicting chronic pain underlines how prolonged emotional stress can trigger central sensitization, heightening the nervous system’s reactivity to pain. These results support a biopsychosocial model in which neuroendocrine alterations, inflammation, emotional dysregulation, and social disconnection converge to maintain or exacerbate chronic pain in this population.

In addition, the data suggest the presence of differentiated pain pathways among victims, shaped by the timing and nature of the trauma experienced. Adult abuse, particularly ongoing psychological violence, appears to contribute to chronic pain via mechanisms such as sustained physiological hyperactivation and emotional dysregulation, whereas childhood abuse may exert long-term effects through developmental disruptions, such as insecure attachment, altered stress appraisal, and heightened sensitivity to future relational trauma.

These differentiated pathways are consistent with developmental psychopathology [64] and cumulative trauma models [65], which emphasize how early adverse experiences interact with later vulnerabilities to shape distinct trajectories of mental and physical health. From this perspective, adult and childhood trauma do not merely add up in their effects but interact with developmental timing, stress system maturation, and interpersonal regulation [66] to produce different patterns of dysregulation and pain chronification. Understanding these trajectories may help identify clinically relevant subtypes of chronic pain and guide personalized intervention strategies.

### 4.5. The Bio-Psycho-Social Risk Factors for Chronic Pain Among Non-Victims

Chronic pain is not exclusive to victims. Although it appears less common among non-victims [25], identifying its risk factors in this group is essential to better understand its broader impact.

Our findings indicate that key bio-psycho-social risk factors for chronic pain in non-victims include muscle inflammation, osteoarthritis, psychological disorders, and low life satisfaction. Among these, muscle inflammation and psychological disorders emerge as the most influential.

Muscle inflammation plays a central role, and studies show a bidirectional relationship with depression: each can intensify the other, creating a feedback loop that aggravates pain symptoms [67]. Similarly, low life satisfaction, often linked to depression, may amplify the perception and persistence of pain [68].

Osteoarthritis also contributes to chronic pain through both physical degeneration and its psychological impact. Inflammatory processes associated with osteoarthritis can worsen emotional distress, which in turn heightens pain sensitivity [69].

In summary, these findings underscore the importance of integrated approaches that address both physical and psychological aspects of chronic pain, even in individuals without a history of victimization.

### 4.6. The Bio-Psycho-Social Risk Factors for Chronic Pain Among Women in General

Men and women exhibit differences in their responses to pain. Bartley et al. [70] found that women are more likely than men to experience chronic pain conditions and tend to report higher pain intensity across various types of pain, which is often attributed to a combination of biological, psychological, and social factors. This highlights the importance of identifying risk factors for chronic pain in women, regardless of victimization status. The current work reveals an intricate interplay between biological, psychological, and social risk factors contributing to chronic pain among women.

Starting with biological factors, studies confirm that urinary tract infections are a significant predictor of chronic pain, often tied to broader inflammatory processes that exacerbate pain sensitivity across various body systems, including the gastrointestinal and reproductive systems. For example, infections like urinary tract infections have been linked to increased chronic pelvic pain and other somatic issues such as skin conditions and cardiovascular symptoms [71].

From a psycho-social perspective, the role of mental health conditions like sleep disorders, mood disorders, and PTSD in chronic pain is well established [54,72]. Psychological distress and somatic symptom disorders amplify pain perception, and PTSD in particular has been shown to sensitize individuals to pain by impacting the nervous system’s stress response mechanisms [73]. Moreover, adverse childhood and adulthood experiences play a critical role in chronic pain development. Trauma can lead to heightened pain sensitivity and an increased likelihood of chronic pain, even without a fully developed psychiatric condition [74].

Interpersonal stressors, such as dysfunctional relationships with family members or the absence of positive parental connections, also contribute to chronic pain risk. Research supports the view that difficult familial relationships can perpetuate cycles of stress and pain through emotional dysregulation and heightened stress response, further embedding these experiences into one’s health outcomes [75]. Additionally, transdiagnostic factors like suicidal ideation, guilt, shame, and high emotional reactivity are often associated with chronic pain, as they intensify emotional suffering and physical exhaustion, creating a self-perpetuating cycle [75]. Together, these results underscore the multifaceted nature of chronic pain and the importance of addressing biological, psychological, and social factors in treatment.

### 4.7. Clinical and Policy Implications

The present findings carry important implications for clinical and policy settings, particularly within forensic contexts and anti-violence services. Given the strong link between recurrent psychological abuse in adulthood and chronic pain, healthcare providers working with survivors, especially in trauma-informed services, should integrate routine assessments for pain symptoms alongside psychological evaluations. Early identification of biological and psychosocial risk factors can facilitate targeted interventions that address both physical and emotional sequelae of abuse. In forensic contexts, recognizing chronic pain as a potential long-term consequence of violence may contribute to more comprehensive evaluations of victim impact and inform appropriate legal and rehabilitative responses. Moreover, anti-violence centers and mental health services could benefit from training that highlights the bio-psycho-social pathways linking trauma and pain, fostering integrated care approaches that include pain management, mental health support, and social resources. Policymakers should also consider incorporating these findings into guidelines that promote interdisciplinary collaboration in supporting survivors. Importantly, the translation of these findings into practice requires ethical sensitivity. Some of the variables identified as risk factors, such as the number of sexual partners or lower educational attainment, may reflect broader social vulnerabilities rather than individual pathology. Without a trauma-informed and contextualized interpretation, the use of such indicators could inadvertently contribute to stigmatization or victim-blaming. It is therefore essential that practitioners are trained to understand these factors within the framework of systemic inequality, gender-based violence, and cumulative trauma. Embedding these findings into screening or assessment protocols should be carried out with caution, ensuring that the rights, dignity, and lived experiences of survivors remain central to any clinical or policy-driven application.

### 4.8. Strengths, Limitations, and Future Directions

This study presents several strengths. It is among the first to investigate and compare biological, psychological, and social risk factors for chronic pain in female victims and non-victims of violence, as well as in women from the general population. By adopting a biopsychosocial framework, the study reflects the complexity of chronic pain and offers clinically relevant insights. Notably, it highlights the role of psychological abuse and trauma history, factors often underexplored in pain research, as significant contributors to chronic pain. The use of real-world data enhances the ecological validity of the findings and supports their clinical and forensic applicability.

However, our study has some limitations. First, to guarantee the anonymity of the victims, we only collected demographic information, specifically age, and scholarships. This limitation prevents us from conducting further analyses. As this is the first study of its kind, additional research is needed, particularly in other countries, to enhance the generalizability of the findings. Then, focusing solely on a female and Italian sample limits our understanding of the situation in the male population. The study performed matching only on age and education level, two important confounding factors, but not on other possible variables that could influence the association between violence and chronic pain. The checklist used to assess risk factors has not yet been validated, although a validation study is currently underway. However, no existing validated tool was available that covers the same range of bio-psycho-social variables relevant to this research aim. Lastly, we acknowledge the possibility of Type I errors due to multiple univariate comparisons. While corrections for multiple testing were not applied to preserve sensitivity in this exploratory phase, this limitation means that some associations may represent false positives.

Future research should aim to validate these findings in larger and more diverse samples, including male and non-binary individuals, and across different cultural contexts. A key next step would be the development and testing of tailored psychological intervention protocols for chronic pain, particularly for women with histories of trauma. These interventions should address trauma-related emotional dysregulation, guilt, and shame, and be informed by trauma-focused and transdaignostic approaches. Longitudinal studies and the inclusion of biological and physiological markers (e.g., HPA axis functioning, inflammatory responses) would also help clarify mechanisms linking trauma and chronic pain.

## 5. Conclusions

Despite some limitations, this case–control study provides valuable insights into a previously unexplored phenomenon while also confirming previous evidence. The findings indicate that exposure to specific bio-psycho-social factors, such as psychological, physical, and sexual violence in adulthood, lipid metabolism disorders, lack of social support, respiratory infections, allergies, anxiety disorders, and having more than four sexual partners in a lifetime, are specific risk factors for the onset of chronic pain in victimized women. These results represent the first investigation into this topic. Additionally, for women in general, the findings affirm the influence of the bio-psycho-social model and are consistent with existing literature. Our results may assist in screening and assessing this unique diagnosis.

## Figures and Tables

**Table 1:** Summary of the ORs.

Risk Factor	OR	95% CI	*p* Fisher’s Exact Test
	Victims		
Lipid metabolism disorder	8.87	1.07–73.5	0.02 *
(Not receiving) Social support	5.37	1.10–26.21	0.03 *
Respiratory infections	4.72	1.21–18.29	0.02 *
Recurrent psychological abuse at adult age	4.40	1.38–14.08	0.01
Anxiety disorder	4.36	1.52–12.51	0.009
Recurrent physical abuse at adult age	4.26	1.09–16.6	0.04 *
Allergies resulting in respiratory symptoms	3.83	0.98–14.99	0.05 *
Single sexual abuse at adult age	3.74	1.09–12.8	0.03 *
More than 4 sexual partners during the course of life	2.92	1.05–8.10	0.04 *
	Non-victims		
Muscle inflammations	18.63	4.00–86.65	<0.001
Any psychological disorder	7.54	1.53–36.99	0.008
Osteoarthritis	6.51	1.30–32.4	0.01
(Dis)Satisfaction for one’s life as it is	2.82	1.17–6.78	0.02 *
	All women		
Vaginal swelling	16.34	0.91–39.1	0.01
Impediment in daily activities	12.3	4.83–31.2	<0.001
Somatizations	7.18	2.80–18.4	<0.001
Urinary infections	6.64	2.17–20.3	<0.001
Suicidal thoughts	6.34	1.77–22.7	0.003
Tiredness/absence of energy	5.77	2.98–11.2	<0.001
Mood disorders	5.36	2.37–12.1	<0.001
PTSD	5.10	2.25–11.56	<0.001
Sleep disorders	4.10	2.11–7.97	<0.001
Heart palpitations	3.91	1.91–8.00	<0.001
(Not having) Good relationships with parents	3.89	1.56–9.69	0.004
Skin problems/irritations	3.22	1.57–6.61	0.002
Recurrent psychological abuse during childhood	3.16	1.31–7.61	0.01
Single physical abuse during childhood	3.15	1.08–9.20	0.04 *
Alterations of intestinal transit	2.64	1.42–4.93	0.003
Somatic disorder	2.46	1.08–5.61	0.04 *
Guilt and shame	2.37	1.25–4.47	0.01
Tense relationships within the current family	2.36	1.19–4.66	0.01
Stress	2.34	1.24–4.41	0.01
Tense relationships within the family of origin	2.15	1.14–4.07	0.02 *
High emotionality	2.13	1.10–4.11	0.03 *
Traumatic experiences during childhood	2.10	1.07–4.11	0.04 *
Traumatic experiences in adulthood	2.04	1.10–3.76	0.03 *

* nominally significant results.

**Table 2:** Binary logistic regression results in victims.

Predictor	S	SE	Z	*p*	OR
Intercept	−5.91	1.71	−3.45	<0.001	0.002
Lipid metabolism disorder	2.79	1.24	2.24	0.02	16.25
Respiratory allergies	1.82	0.80	2.26	0.02	6.19
Recurrent psychological abuse at adult age	1.54	0.71	2.15	0.03	4.66
Single sexual abuse at adult age	1.50	0.72	2.09	0.03	4.50
**Model Fit**	**R^2^N**	**χ^2^**	**DF**	***p***	**AUC**
	0.38	22.6	4	<0.001	0.80

**Table 3:** Binary logistic regression results in non-victims.

Predictor	S	SE	Z	*p*	OR
Intercept	−3.59	1.10	−3.24	0.001	0.02
Muscle inflammations	2.80	0.79	3.52	<0.001	16.39
Any psychological disorders	1.72	0.87	1.97	0.04	5.58
**Model Fit**	**R^2^N**	**χ^2^**	**DF**	***p***	**AUC**
	0.31	27.3	2	<0.001	0.71

**Table 4:** Binary logistic regression results for women.

Predictor	S	SE	Z	*p*	OR
Intercept	−4.18	0.82	−5.08	<0.001	0.01
Urinary infections	1.90	0.64	2.94	0.003	6.71
Impediments in daily activities	1.86	0.51	3.61	<0.001	6.45
Tiredness	1.01	0.38	2.61	0.009	2.75
Sleep disorders	0.89	0.40	2.18	0.02	2.43
**Model Fit**	**R^2^N**	**χ^2^**	**DF**	***p***	**AUC**
	0.42	64.2	4	<0.001	0.82

## Data Availability

The authors confirm that the data supporting the findings of this study are available within the article and its Supplementary Materials.

## Supplementary Materials

The following supporting information can be downloaded at: https://www.mdpi.com/article/10.3390/life15060976/s1, Table S1: Checklist; Table S2: OR and logistic regression complete results. Supplementary material includes the complete checklist in Italian, English, and Spanish, as well as the full odds ratios and logistic regression results, not just the significant ones.
