# Supplementary material for: Risk Factors for Chronic Pain in Women: The Role of Violence Exposure in a Case–Control Study
Source: Life (Basel). 2025 Jun 18;15(6):976. doi: 10.3390/life15060976 (PMC12194286; doi:10.3390/life15060976)
Supplement: Supplementary file 1 [file life-15-00976-s001.zip › life-3635769-supplementary.pdf]

Supplementary Materials

Table S1. Checklist in Italian, English, and Spanish (for English or Spanish validation contact the corresponding author)

**Categorie BIO-PSICO-SOCIALI - BIO-PSYCHO-SOCIAL Categories - Categorías BIO-PSICO-SOCIAL**	**SI/YES**	**NO**
Sovrappeso/obesità - Overweight/obesity - Sobrepeso/obesidad		
Sottopeso - Underweight - Bajo peso		
Infezioni delle vie respiratorie ricorrenti (sinusiti, congestioni nasali) - Recurrent respiratory infections (sinusitis, nasal congestion) - Infecciones recurrentes del tracto respiratorio (sinusitis, congestión nasal)		
Allergie che comportano sintomi respiratori (asma, rinite allergica) - Allergies resulting in respiratory symptoms (asthma, allergic rhinitis) - Alergias que provoquen síntomas respiratorios (asma, rinitis alérgica)		
Infezioni sessualmente trasmissibili (AIDS, clamidia) - Transmissible sexual infections (AIDS, chlamydia) - Infecciones de transmisión sexual (SIDA, clamidia)		
Infezioni delle vie urinarie ricorrenti (cistiti, uretriti, pielonefriti) - Recurrent urinary infections (cystitis, urethritis, pyelonephritis) - Infecciones recurrentes del tracto urinario (cistitis, uretritis, pielonefritis)		
Infezioni ginecologiche ricorrenti (vaginiti, vulviti, cerviciti) - Recurrent gynecological infections (vaginitis, vulvitis, cervicitis) - Infecciones ginecológicas recurrentes (vaginitis, vulvitis, cervicitis)		
Rigonfiamento vaginale - Vaginal swelling - Hinchazón vaginal		
Perdite urinarie ricorrenti - Recurrent urine leakage - Fugas urinarias recurrentes		
Vescicole/ulcere genitali - Genital vesicles/ulcers - Vesículas/úlceras genitales		
Sanguinamento ricorrente dopo i rapporti sessuali - Recurrent bleeding after sexual intercourse - Sangrado recurrente después de las relaciones sexuales		
Prolasso uterovaginale - Uterovaginal prolapse - Prolapso uterovaginal		
Ipertensione arteriosa (pressione alta) - Arterial hypertension (high blood pressure) - Hipertensión arterial (presión arterial alta)		
Palpitazioni cardiache - Heart palpitations - Palpitaciones del corazón		
Disturbo del metabolismo dei lipidi - Lipid metabolism disorder - Trastorno del metabolismo de los lípidos		
Diabete - Diabetes - Diabetes		

Ipercolesterolemia - Hypercholesterolemia - Hipercolesterolemia		
Patologie tiroidee (ipertiroidismo, ipotiroidismo) - Thyroid pathologies (hyperthyroidism, hypothyroidism) - Patologías tiroideas (hipertiroidismo, hipotiroidismo)		
Sintomi della menopausa intensi (vampate di calore, sudorazione, secchezza vaginale, osteoporosi) - Intense menopause symptoms (hot flushes, sweating, vaginal dryness, osteoporosis) - Síntomas intensos de menopausia (sofocos, sudoración, sequedad vaginal, osteoporosis)		
Ciclo mestruale irregolare/assente - Irregular/absent menstrual cycle - Ciclo menstrual irregular/sin período		
Reflusso gastro-esofageo - Gastroesophageal reflux -Reflujo gastroesofágico		
Alterazioni ricorrenti nel transito intestinale (stitichezza, costipazione, diarrea) - Recurrent alterations of intestinal transit (constipation, diarrhoea) - Alteraciones recurrentes en el tránsito intestinal (estreñimiento, diarrea)		
Problemi/irritazioni cutanee (dermatite, eczema, rash) - Skin problems/irritations (dermatitis, ecxema, rush) - Problemas/irritaciones de la piel (dermatitis, eczema, erupción)		
Otiti/congiuntiviti ricorrenti - Recurrent otitis/conjunctivitis - Infecciones de oído/conjuntivitis recurrentes		
Infiammazione muscolare - Muscle inflammation - Inflamación muscular		
Osteoartrite - Osteoarthritis - Osteoartritis		
Disturbi del sonno (insonnia, ipersonnia) - Sleep disorders (insomnia, hypersomnia) - Trastornos del sueño (insomnio, hipersomnia)		
Disturbo d'ansia (disturbo di panico, disturbo di ansia sociale, disturbo d'ansia generalizzato, fobia specifica) - Anxiety disorder (panic disorder, social anxiety disorder, generalized anxiety disorder, specific phobia) - Trastorno d'ansiedad (trastorno de pánico, trastorno de ansiedad social, d'ansiedad generalizada, fobia específica)		
Disturbo dell'umore (depressione, disturbo bipolare) - Mood disorder (depression, bipolar disorder) - Perturbación de'estado de ánimo (depresión, trastorno bipolar)		
Disturbo da stress post-traumatico - Post-Traumatic stress disorder - Trastorno de estrés postraumático		
Disturbo somatico (sintomi somatici, ipocondria, disturbo di conversione) - Somatic disorder (somatic symptoms, hypochondria, convertion disorder) - Trastorno somático (síntomas somáticos, hipocondría, trastorno de conversión)		
Richiesta di parere medico per un disturbo senza poi ricevere assistenza per quello - Request for a medical advice for a disorder without receiving assistance for it - Solicitar consejo médico para un trastorno sin recibir luego asistencia para ello		

Disturbo alimentare (anoressia, bulimia, binge eating) - Eating disorder (anorexia, bulimia, binge eating) - Trastorno alimentario (anorexia, bulimia, atracones)		
Diagnosi di qualsiasi disturbo psicologico - Diagnosis of any psychological disorder - Diagnóstico de cualquier trastorno psicológico		
Assunzione di sostanze psicoattive - Psychoactive substance intake - Ingesta de sustancias psicoactivas		
Più di 3 assunzioni di alcool durante la settimana - More then 3 alcohol assumption during the week - Mas que 3 ingestas de alcohol durante la semana		
Condizione di fumatrice - Smoker - Fumo de cigarros		
Singolo abuso sessuale da parte del partner durante l'età adulta (>18 anni) - Single sexual abuse by your partner at adult age (>18 years) - Abuso sexual individual por parte de la pareja durante su edad adulta (>18 años)		
Ricorrenti abusi sessuali da parte del partner durante l'età adulta (>18 anni) - Recurrent sexual abuse by your partner at adult age (>18 years) - Abuso sexual recurrente por parte de la pareja durante su edad adulta (>18 años)		
Singolo abuso fisico da parte del partner durante l'età adulta (>18 anni) - Single physical abuse by your partner at adult age (>18 years) - Abuso físico único por parte de la pareja durante su edad adulta (>18 años)		
Ricorrenti abusi fisici da parte del partner durante l'età adulta (>18 anni) - Recurrent physical abuse by your partner at adult age (>18 years) - Abuso físico recurrente por parte de la pareja durante el embarazo (>18 años)		
Singolo abuso psicologico da parte del partner durante l'età adulta (>18 anni) - Single psychological abuse by your partner at adult age (>18 years) - Maltrato psicológico único por parte de la pareja durante su edad adulta(>18 años)		
Ricorrenti abusi psicologici da parte del partner durante l'età adulta (>18 anni) - Recurrent psychological abuse by your partner at adult age (>18 years) - Abuso psicológico recurrente por parte de la pareja durante su edad adulta (>18 años)		
Singolo abuso sessuale durante l'infanzia (< 18 anni) - Single sexual abuse during childhood (<18 years) - Abuso sexual único durante infancia (< 18 años)		
Ricorrenti abusi sessuali durante l'infanzia (< 18 anni) - Recurrent sexual abuse during childhood (<18 years) - Abuso sexual recurrente durante infancia (< 18 años)		
Singolo abuso fisico durante l'infanzia (< 18 anni) - Single physical abuse during childhood (<18 years) - Abuso físico único durante infancia (< 18 años)		
Ricorrenti abusi fisici durante l'infanzia (< 18 anni) - Recurrent physical abuse during childhood (<18 years) - Abuso físico recurrente durante infancia (< 18 años)		
Singolo abuso psicologico durante l'infanzia (< 18 anni) - Single psychological abuse during childhood (<18 years) - Maltrato psicológico único durante infancia (< 18 años)		

Ricorrenti abusi psicologici durante l'infanzia (< 18 anni) - Recurrent psychological abuse during childhood (<18 years) - Abuso psicológico recurrente durante infancia (< 18 años)		
Esperienze traumatiche durante l'infanzia subite o assistite (lutti, incidenti, violenza assistita, gravi malattie, guerra) - Traumatic experiences during childhood endured or witnessed (be reavement, accidents, assisted violence, serious illnesses, war) - Experiencias traumáticas durante infancia sufrida o presenciada (duelo, accidentes, violencia presenciada, enfermedades graves, guerra)		
Esperienze traumatiche durante l'età adulta subite o assistite (lutti, incidenti, violenza, gravi malattie, guerra) - Traumatic experiences in adulthood endured or witnessed (be reavement, accidents, violence, serious illnesses, war) - Experiencias traumáticas durante edad adulta sufrido o presenciado en la edad adulta (muerte, accidentes, violencia presenciada, enfermedades graves, guerra)		
Patologie psichiatriche in famiglia (madre, padre, zii, nonni) - Psychiatric diseases in the family (mother, father, uncles, grandparents) - Patologías psiquiátricas en la familia (madre, padre, tíos, abuelos)		
Rapporti tesi all'interno della famiglia di origine - Tense relationships within the family of origin - Relaciones tensas dentro de la familia de origen		
Rapporti tesi all'interno dell'attuale nucleo familiare - Tense relationships within the current family unit - Relaciones tensas en todo el interior de la unidad familiar actual		
Buon rapporto con i genitori - Good relationship with parents - Buena relación con los padres		
Storia di aborto/i spontaneo/i - History of spontaneous abortion(s) - Historial de abortos espontáneos		
Storia di interruzione volontaria di gravidanza - History of voluntary termination of pregnancy - Historia de interrupción voluntaria del embarazo		
Soddisfazione per la propria vita così come è - Satisfaction for one's life as it is - Satisfacción con la vida tal como es		
Elevati livelli di stress - High stress levels - Altos niveles de estrés		
Ricorrenti pensieri suicidari - Recurrent suicidal thoughts - Pensamientos suicidas recurrentes		
Ricorrenti sentimenti di colpa e vergogna - Recurring feelings of guilt and shame - Sentimientos recurrentes de culpa y vergüenza		
Buona autostima - Good self-esteem - Buena autoestima		
Rapporti sessuali soddisfacenti - Satisfactory sexual intercourse - Relaciones sexuales satisfactorias		
Desiderio sessuale - Sexual desire - Deseo sexual		
Amicizie significative - Meaningful friendships - Amistades significativas		

Ottenimento di supporto in caso di bisogno - Getting support in case of need - Obtener apoyo cuando sea necesario		
Impedimento nello svolgimento di normali attività quotidiane a causa di dolore - Impediment in carrying out normal daily activities due to pain - Impedimento para realizar actividades normales adiariamente debido al dolor		
Attività fisica/sport regolare - Regular physical activity/sport - Actividad física/deporte regular		
Stanchezza/assenza di energie anche di prima mattina - Tiredness/absence of energy even in the early morning - Cansancio/falta de energía incluso temprano en la mañana		
Elevata emotività (espressione ed esperienza degli stati emotivi molto intensa) - High emotionality (very intense expression and experience of emotional states) - Alta emocionalidada (expresión y experiencia muy intensa de estados emocionales)		
Più di 4 partner sessuali durante il corso della vita - More than 4 sexual partners during the course of life - Mas que 4 parejas sexuales a lo largo de la vida		
Età del primo rapporto sessuale inferiore a 14 anni - Age of first sexual intercourse less than 14 years - Primera relación sexual antes de los 14 años		
Presenza di dolore durante il rapporto sessuale - Presence of pain during sexual intercourse - Presencia de dolor durante las relaciones sexuales		

Table S2. OR and logistic regression complete results

Risk factor	Victims/Non Victims x Cases/Controls	OR	95% level of confidence - lower	95% level of confidence - upper	p Fisher's exact test	p Logistic Regression
Overweight/Obesity	• Victims: 19/49<br>• Non-victims: 29/73<br>• Total: 48/122	• 1.09<br>• 2.10<br>• 1.60	• 0.36<br>• 0.87<br>• 0.81	• 3.25<br>• 5.02<br>• 3.14	• 1.00<br>• 0.12<br>• 0.23	• 0.88<br>• 0.09<br>• 0.17
Underweight	• Victims: 12/56<br>• Non-victims: 2/100<br>• Total: 14/156	• 0.84<br>• 7.16*<br>• 1.89	• 0.23<br>• 0.33<br>• 0.60	• 2.99<br>• 153.22<br>• 5.91	• 1.00<br>• 0.17<br>• 0.40	• 0.78<br>• 0.98<br>• 0.27

Respiratory infections (sinusitis, nasal congestion)	• Victims: 19/49<br>• Non-victims: 18/84<br>• Total: 37/133	• 4.72<br>• 2.55<br>• 3.49	• 1.21<br>• 0.89<br>• 1.56	• 18.29<br>• 7.26<br>• 7.79	• 0.02<br>• 0.11<br>• 0.003	• 0.02<br>• 0.07<br>• 0.002
Allergies resulting in respiratory symptoms (asthma, allergic rhinitis)	• Victims: 17/51<br>• Non-victims: 17/85<br>• Total: 35/136	• 3.83<br>• 0.95<br>• 1.81	• 0.98<br>• 0.33<br>• 0.84	• 14.99<br>• 2.74<br>• 3.92	• 0.05<br>• 1.00<br>• 0.17	• 0.05<br>• 0.92<br>• 0.12
Urinary infections (cystitis, urethritis, pyelonephritis)	• Victims: 17/51<br>• Non-victims: 8/94<br>• Total: 25/145	• 3.83<br>• 11.28<br>• 6.64	• 0.98<br>• 1.33<br>• 2.17	• 15<br>• 95.5<br>• 20.3	• 0.05<br>• 0.009<br>• <.001	• 0.05<br>• 0.02<br>• <.001
Gynecological infections (vaginitis, vulvitis, cervicitis)	• Victims: 19/49<br>• Non-victims: 7/95<br>• Total: 26/144	• 2.10<br>• 1.03<br>• 2.11	• 0.65<br>• 0.21<br>• 0.88	• 6.75<br>• 4.87<br>• 5.05	• 0.27<br>• 1.00<br>• 0.13	• 0.21<br>• 0.96<br>• 0.09
Vaginal swelling	• Victims: 6/62<br>• Non-victims: 9/93<br>• Total: 26/144	• 9.44*<br>• 4.20*<br>• 16.34*	• 0.50<br>• 0.16<br>• 0.91	• 175<br>• 106<br>• 39.1	• 0.07<br>• 0.42<br>• 0.01	• 0.09<br>• 0.09<br>• 0.05
Urine leakage	• Victims: 17/51<br>• Non-victims: 9/93<br>• Total: 26/144	• 1.68<br>• 1.81<br>• 2.11	• 0.51<br>• 0.45<br>• 0.88	• 5.48<br>• 7.18<br>• 5.05	• 0.56<br>• 0.48<br>• 0.13	• 0.39<br>• 0.39<br>• 0.09
Genital vesicles/ulcers	• Victims: 2/66<br>• Non-victims: 1/101<br>• Total: 3/167	• 0.15*<br>• 0.01*<br>• 0.18	• 0.15<br>• 0.01<br>• 0.18	• 70.9<br>• 11.3<br>• 22.8	• 0.52<br>• 1.00<br>• 1.00	• 0.99<br>• 0.99<br>• 0.56
Bleeding after sexual intercourse	• Victims: 3/65<br>• Non-victims: 2/100<br>• Total: 5/165	• 4.70*<br>• 1.38<br>• 4.15	• 0.23<br>• 0.08<br>• 0.45	• 94.7<br>• 22.7<br>• 37.9	• 0.28<br>• 1.00<br>• 0.36	• 0.99<br>• 0.82<br>• 0.20

Uterovaginal prolapse	• Victims:8/60<br>• Non-victims: 4/98<br>• Total: 12/158	• 1.04<br>• 1.39<br>• 1.44	• 0.22<br>• 0.18<br>• 0.43	• 4.75<br>• 10.28<br>• 4.72	• 1.00<br>• 1.00<br>• 0.76	• 0.96<br>• 0.74<br>• 0.55
Arterial hypertension	• Victims: 7/61<br>• Non-victims: 14/88<br>• Total: 21/149	• 1.62<br>• 1.44<br>• 1.39	• 0.29<br>• 0.46<br>• 0.55	• 9.04<br>• 4.47<br>• 3.49	• 0.70<br>• 0.56<br>• 0.64	• 0.58<br>• 0.52<br>• 0.48
Heart palpitations	• Victims: 28/40<br>• Non-victims: 23/79<br>• Total: 51/119	• 3.67<br>• 3.42<br>• 3.91	• 1.23<br>• 1.29<br>• 1.91	• 10.96<br>• 9.05<br>• 8.00	• 0.02<br>• 0.01<br>• <.001	• 0.02<br>• 0.01<br>• <.001
Lipid metabolism disorder	• Victims: 12/56<br>• Non-victims: 3/99<br>• Total: 15/155	• 8.87<br>• 2.83<br>• 7.49	• 1.07<br>• 0.24<br>• 1.63	• 73.5<br>• 32.3<br>• 34.3	• 0.02<br>• 0.57<br>• 0.005	• 0.04<br>• 0.40<br>• 0.009
Diabetes	• Victims: 3/65<br>• Non-victims: 2/100<br>• Total: 5/165	• 0.29<br>• 1.38<br>• 0.65	• 0.02<br>• 0.08<br>• 0.10	• 3.40<br>• 22.71<br>• 4.04	• 0.55<br>• 1.00<br>• 1.00	• 0.32<br>• 0.82<br>• 0.65
Hypercholesterolemia	• Victims: 12/56<br>• Non-victims: 13/89<br>• Total: 25/145	• 2.09<br>• 0.57<br>• 1.09	• 0.51<br>• 0.16<br>• 0.47	• 8.57<br>• 1.99<br>• 2.57	• 0.34<br>• 0.54<br>• 1.00	• 0.30<br>• 0.37<br>• 0.82
Thyroid pathologies (hyperthyroidism, hypothyroidism)	• Victims: 16/52<br>• Non-victims: 14/88<br>• Total: 30/140	• 3.43<br>• 0.32<br>• 1.17	• 0.87<br>• 0.08<br>• 0.53	• 13.52<br>• 1.25<br>• 2.59	• 0.08<br>• 0.14<br>• 0.84	• 0.07<br>• 0.10<br>• 0.68
Intense menopause symptoms (hot flushes, sweating, vaginal dryness, osteoporosis)	• Victims: 15/53<br>• Non-victims: 18/84<br>• Total: 33/137	• 1.95<br>• 1.12<br>• 1.46	• 0.54<br>• 0.40<br>• 0.67	• 6.94<br>• 3.12<br>• 3.14	• 0.37<br>• 1.00<br>• 0.43	• 0.30<br>• 0.82<br>• 0.33

Irregular/absent menstrual cycle	• Victims: 28/40<br>• Non-victims: 41/61<br>• Total: 69/101	• 0.55<br>• 1.33<br>• 0.95	• 0.20<br>• 0.59<br>• 0.51	• 1.50<br>• 2.96<br>• 1.76	• 0.31<br>• 0.54<br>• 1.00	• 0.24<br>• 0.48<br>• 0.87
Gastroesophageal reflux	• Victims: 26/42<br>• Non-victims: 31/71<br>• Total: 57/113	• 1.69<br>• 1.19<br>• 1.45	• 0.60<br>• 0.50<br>• 0.76	• 4.74<br>• 2.79<br>• 2.75	• 0.44<br>• 0.82<br>• 0.33	• 0.32<br>• 0.68<br>• 0.25
Recurrent alterations of intestinal transit (constipation, diarrhea)	• Victims: 34/34<br>• Non-victims: 42/60<br>• Total: 76/94	• 2.78<br>• 2.42<br>• 2.64	• 1.01<br>• 1.08<br>• 1.42	• 7.67<br>• 5.45<br>• 4.93	• 0.08<br>• 0.04<br>• 0.003	• 0.04<br>• 0.03<br>• 0.002
Skin problems/irritations (dermatitis, eczema, rash)	• Victims: 26/42<br>• Non-victims: 21/81<br>• Total: 47/123	• 3.03<br>• 2.76<br>• 3.22	• 1.01<br>• 1.03<br>• 1.57	• 9.06<br>• 7.43<br>• 6.61	0.07<br>0.04<br>0.002	• 0.04<br>• 0.04<br>• 0.001
Otitis/conjunctivitis	• Victims: 5/63<br>• Non-victims: 4/98<br>• Total: 9/161	• 2.63<br>• 1.39<br>• 2.08	• 0.27<br>• 0.18<br>• 0.50	• 24.93<br>• 10.28<br>• 8.59	• 0.64<br>• 1.00<br>• 0.49	• 0.39<br>• 0.74<br>• 0.31
Muscle inflammations	• Victims: 27/41<br>• Non-victims: 19/83<br>• Total: 46/124	• 2.47<br>• 18.63<br>• 6.51	• 0.85<br>• 4.00<br>• 2.88	• 7.10<br>• 86.65<br>• 14.68	0.12<br><.001<br><.001	• 0.09<br>• <.001<br>• <.001
Osteoarthritis	• Victims: 13/55<br>• Non-victims: 19/92<br>• Total: 23/147	• 4.26<br>• 6.51<br>• 5.83	• 0.86<br>• 1.30<br>• 1.89	• 21.1<br>• 32.4<br>• 18.0	0.11<br>0.01<br>0.001	• 0.07<br>• 0.02<br>• 0.002
Sleep disorders (insomnia, hypersomnia)	• Victims: 38/30<br>• Non-victims: 27/75<br>• Total: 65/105	• 3.20<br>• 4.00<br>• 4.10	• 1.16<br>• 1.57<br>• 2.11	• 8.85<br>• 10.17<br>• 7.97	• 0.02<br>• 0.003<br>• <.001	• 0.02<br>• 0.004<br>• <.001

Anxiety disorder (panic disorder, social anxiety disorder, generalized anxiety disorder, specific phobia)	• Victims: 43/25<br>• Non-victims: 31/71<br>• Total: 74/96	• 4.36<br>• 1.74<br>• 2.94	• 1.52<br>• 0.74<br>• 1.56	• 12.51<br>• 4.07<br>• 5.53	0.009<br>0.27<br>0.001	• 0.006<br>• 0.20<br>• <.001
Mood disorder (depression, bipolar, disorder)	• Victims: 26/42<br>• Non-victims: 16/86<br>• Total: 42/128	• 4.20<br>• 5.32<br>• 5.36	• 1.33<br>• 1.58<br>• 2.37	• 13.2<br>• 17.9<br>• 12.1	0.02<br>0.005<br><.001	• 0.01<br>• 0.007<br>• <.001
PTSD	• Victims: 35/33<br>• Non-victims: 6/96<br>• Total: 41/129	• 3.07<br>• 6.78*<br>• 5.10	• 1.11<br>• 1.13<br>• 2.25	• 8.51<br>• 376.87<br>• 11.56	0.04<br>0.005<br><.001	• 0.03<br>• 0.99<br>• <.001
Somatic disorder (somatic symptoms, hypochondria, conversion disorder)	• Victims: 20/48<br>• Non-victims: 11/91<br>• Total: 31/139	• 1.67<br>• 2.67<br>• 2.46	• 0.54<br>• 0.73<br>• 1.08	• 5.08<br>• 9.79<br>• 5.61	0.42<br>0.19<br>0.04	• 0.36<br>• 0.13<br>• 0.03
Somatizations	• Victims: 24/44<br>• Non-victims: 12/90<br>• Total: 36/134	• 5.00<br>• 8.64<br>• 7.18	• 1.47<br>• 1.78<br>• 2.80	• 17.0<br>• 41.8<br>• 18.4	0.009<br>0.004<br><.001	• 0.01<br>• 0.007<br>• <.001
Eating disorder (anorexia, bulimia, binge eating)	• Victims: 16/52<br>• Non-victims: 7/95<br>• Total: 23/147	• 1.04<br>• 1.91<br>• 1.67	• 0.32<br>• 0.40<br>• 0.67	• 3.31<br>• 9.04<br>• 4.09	• 1.00<br>• 0.45<br>• 0.37	• 0.94<br>• 0.41<br>• 0.26
Any psychological disorder	• Victims: 19/49<br>• Non-victims: 11/91<br>• Total: 30/140	• 1.49<br>• 7.54<br>• 3.36	• 0.48<br>• 1.53<br>• 1.40	• 4.59<br>• 36.99<br>• 8.06	0.58<br>0.008<br>0.008	• 0.48<br>• 0.01<br>• 0.007

Psychoactive substance intake	• Victims: 13/55<br>• Non-victims: 4/98<br>• Total: 17/153	• 0.66<br>• 4.35<br>• 1.48	• 0.19<br>• 0.43<br>• 0.53	• 2.26<br>• 43.33<br>• 4.11	• 0.54<br>• 0.30<br>• 0.61	• 0.51<br>• 0.21<br>• 0.44
Alcohol consumption	• Victims: 7/61<br>• Non-victims: 10/92<br>• Total: 17/153	• 0.66<br>• 0.14<br>• 0.67	• 0.19<br>• 0.007<br>• 0.24	• 2.26<br>• 2.70<br>• 1.86	• 0.54<br>• 0.13<br>• 0.61	• 0.51<br>• 0.98<br>• 0.44
Smoker	• Victims: 31/37<br>• Non-victims: 23/79<br>• Total: 54/116	• 1.24<br>• 1.69<br>• 1.73	• 0.46<br>• 0.66<br>• 0.89	• 3.32<br>• 4.30<br>• 3.33	• 0.80<br>• 0.33<br>• 0.13	• 0.66<br>• 0.27<br>• 0.10
Single sexual abuse at adult age (>18 years)	• Victims: 21/47<br>• Non-victims: 0/102<br>• Total: 21/149	• 3.74<br>• 1.37*<br>• 5.06	• 1.09<br>• nc<br>• 1.63	• 12.8<br>• nc<br>• 15.8	• 0.03<br>• 1.00<br>• 0.004	• 0.03<br>• nc<br>• 0.005
Recurrent sexual abuse at adult age (>18 years)	• Victims: 11/57<br>• Non-victims: 0/102<br>• Total: 11/159	• 1.80<br>• 1.37*<br>• 2.84	• 0.43<br>• nc<br>• 0.72	• 7.53<br>• nc<br>• 11.10	• 0.51<br>• 1.00<br>• 0.21	• 0.41<br>• nc<br>• 0.13
Single physical abuse at adult age (>18 years)	• Victims: 21/47<br>• Non-victims: 0/102<br>• Total: 21/149	• 1.01<br>• 1.37*<br>• 1.74	• 0.350<br>• nc<br>• 0.681	• 2.91<br>• nc<br>• 4.44	• 1.00<br>• 1.00<br>• 0.35	• 0.98<br>• nc<br>• 0.24
Recurrent physical abuse at adult age (>18 years)	• Victims: 18/50<br>• Non-victims: 0/102<br>• Total: 18/152	• 4.26<br>• 1.37*<br>• 5.86	• 1.09<br>• nc<br>• 1.63	• 16.6<br>• nc<br>• 21.1	• 0.04<br>• 1.00<br>• 0.005	• 0.03<br>• nc<br>• 0.007
Single psychological abuse at adult age (>18 years)	• Victims: 26/42<br>• Non-victims: 0/102<br>• Total: 26/144	• 0.75<br>• 1.37*<br>• 1.44	• 0.27<br>• nc<br>• 0.62	• 2.06<br>• nc<br>• 3.35	• 0.61<br>• 1.00<br>• 0.52	• 0.58<br>• nc<br>• 0.39

Recurrent psychological abuse at adult age (>18 years)	• Victims: 51/17<br>• Non-victims: 0/102<br>• Total: 51/119	• 4.40<br>• 1.37*<br>• 3.43	• 1.38<br>• nc<br>• 1.70	• 14.08<br>• nc<br>• 6.93	• 0.01<br>• 1.00<br>• <.001	• 0.01<br>• nc<br>• <.001
Single sexual abuse during childhood (<18 years)	• Victims: 12/56<br>• Non-victims: 0/102<br>• Total: 12/158	• 0.84<br>• 1.37*<br>• 1.43	• 0.23<br>• nc<br>• 0.43	• 2.99<br>• nc<br>• 4.72	• 1.00<br>• 1.00<br>• 0.76	• 0.78<br>• nc<br>• 0.55
Recurrent sexual abuse during childhood (<18 years)	• Victims: 3/65<br>• Non-victims: 0/102<br>• Total: 3/167	• 1.25<br>• 1.37*<br>• 2.02	• 0.10<br>• nc<br>• 0.18	• 14.5<br>• nc<br>• 22.8	• 1.00<br>• 1.00<br>• 1.00	• 0.85<br>• nc<br>• 0.56
Single physical abuse during childhood (<18 years)	• Victims: 19/49<br>• Non-victims: 0/102<br>• Total: 19/151	• 2.10<br>• 1.37*<br>• 3.15	• 0.65<br>• nc<br>• 1.08	• 6.75<br>• nc<br>• 9.20	• 0.27<br>• 1.00<br>• 0.04	• 0.21<br>• nc<br>• 0.03
Recurrent physical abuse during childhood (<18 years)	• Victims: 12/56<br>• Non-victims: 0/102<br>• Total: 12/158	• 2.09<br>• 1.37*<br>• 3.24	• 0.51<br>• nc<br>• 0.84	• 8.57<br>• nc<br>• 12.40	• 0.34<br>• 1.00<br>• 0.13	• 0.30<br>• nc<br>• 0.08
Single psychological abuse during childhood (<18 years)	• Victims: 22/46<br>• Non-victims: 0/102<br>• Total: 22/148	• 0.64<br>• 1.37*<br>• 1.23	• 0.22<br>• nc<br>• 0.50	• 1.80<br>• nc<br>• 3.03	• 0.43<br>• 1.00<br>• 0.82	• 0.39<br>• nc<br>• 0.64
Recurrent psychological abuse during childhood (<18 years)	• Victims: 29/39<br>• Non-victims: 0/102<br>• Total: 29/141	• 2.25<br>• 1.37*<br>• 3.16	• 0.80<br>• nc<br>• 1.31	• 6.30<br>• nc<br>• 7.61	• 0.13<br>• 1.00<br>• 0.01	• 0.12<br>• nc<br>• 0.01

Traumatic experiences during childhood endured or witnessed (bereavement, accidents, witnessed violence, serious illnesses, war)	• Victims: 36/32<br>• Non-victims: 15/87<br>• Total: 51/119	• 2.01<br>• 1.24<br>• 2.10	• 0.74<br>• 0.41<br>• 1.07	• 5.41<br>• 3.73<br>• 4.11	0.21<br>0.78<br>0.04	• 0.16<br>• 0.70<br>• 0.03
Traumatic experiences in adulthood endured or witnessed (bereavement, accidents, witnessed violence, serious illnesses, war)	• Victims: 51/17<br>• Non-victims: 32/70<br>• Total: 83/87	• 1.18<br>• 1.92<br>• 2.04	• 0.38<br>• 0.82<br>• 1.10	• 3.61<br>• 4.47<br>• 3.76	0.78<br>0.13<br>0.03	• 0.77<br>• 0.13<br>• 0.02
Psychiatric diseases in the family (mother, father, uncles, grandparents)	• Victims: 24/44<br>• Non-victims: 15/87<br>• Total: 39/131	• 1.38<br>• 0.90<br>• 1.39	• 0.48<br>• 0.29<br>• 0.68	• 3.92<br>• 2.75<br>• 2.87	• 0.60<br>• 1.00<br>• 0.46	• 0.54<br>• 0.85<br>• 0.36
Tense relationships within the family of origin	• Victims: 40/28<br>• Non-victims: 23/79<br>• Total: 63/107	• 2.33<br>• 1.35<br>• 2.15	• 0.85<br>• 0.52<br>• 1.14	• 6.36<br>• 3.42<br>• 4.07	0.12<br>0.63<br>0.02	• 0.09<br>• 0.53<br>• 0.01
Tense relationships within the current family	• Victims: 40/28<br>• Non-victims: 11/91<br>• Total: 51/119	• 2.33<br>• 1.16<br>• 2.36	• 0.85<br>• 0.33<br>• 1.19	• 6.36<br>• 4.09<br>• 4.66	0.12<br>1.00<br>0.01	• 0.09<br>• 0.81<br>• 0.01
Good relationship with parents	• Victims: 45/23<br>• Non-victims: 96/6<br>• Total: 141/29	• 3.15<br>• 2.92<br>• 3.89	• 0.99<br>• 0.05<br>• 1.56	• 9.95<br>• 16.7<br>• 9.69	0.06<br>0.23<br>0.004	• 0.05<br>• 0.22<br>• 0.004
History of spontaneous abortion(s)	• Victims: 10/58<br>• Non-victims: 12/90<br>• Total: 22/148	• 2.82<br>• 0.97<br>• 1.52	• 0.55<br>• 0.28<br>• 0.61	• 14.49<br>• 3.32<br>• 3.78	• 0.29<br>• 1.00<br>• 0.49	• 0.21<br>• 0.97<br>• 0.36

History of voluntary termination of pregnancy	• Victims: 10/58<br>• Non-victims: 6/96<br>• Total: 16/154	• 0.56<br>• 0.25<br>• 0.57	• 0.14<br>• 0.02<br>• 0.19	• 2.19<br>• 2.29<br>• 1.64	• 0.48<br>• 0.39<br>• 0.43	• 0.41<br>• 0.22<br>• 0.29
Satisfaction for one's life as it is	• Victims: 20/48<br>• Non-victims: 72/30<br>• Total: 92/78	• 2.68<br>• 2.82<br>• 3.22	• 0.92<br>• 1.17<br>• 1.71	• 7.85<br>• 6.78<br>• 6.05	0.10<br>0.02<br><.001	• 0.07<br>• 0.02<br>• <.001
Stress	• Victims: 50/18<br>• Non-victims: 53/49<br>• Total: 103/67	• 1.94<br>• 2.14<br>• 2.34	• 0.65<br>• 0.95<br>• 1.24	• 5.79<br>• 4.79<br>• 4.41	0.26<br>0.07<br>0.01	• 0.23<br>• 0.06<br>• 0.008
Suicidal thoughts	• Victims: 16/52<br>• Non-victims: 3/99<br>• Total: 19/151	• 3.44<br>• 10.28*<br>• 6.34	• 0.87<br>• 0.51<br>• 1.77	• 13.5<br>• 204.5<br>• 22.7	0.08<br>0.07<br>0.003	• 0.07<br>• 0.99<br>• 0.004
Feelings of guilt and shame	• Victims: 44/24<br>• Non-victims: 21/81<br>• Total: 65/105	• 1.64<br>• 2.15<br>• 2.37	• 0.59<br>• 0.81<br>• 1.25	• 4.52<br>• 5.69<br>• 4.47	0.43<br>0.14<br>0.01	• 0.34<br>• 0.12<br>• 0.008
Good self-estem	• Victims: 28/40<br>• Non-victims: 65/37<br>• Total: 93/77	• 1.20<br>• 0.93<br>• 0.86	• 0.44<br>• 0.41<br>• 0.47	• 3.26<br>• 2.11<br>• 1.59	• 0.80<br>• 1.00<br>• 0.75	• 0.72<br>• 0.86<br>• 0.64
Satisfactory sexual intercourse	• Victims: 29/39<br>• Non-victims: 67/35<br>• Total: 96/74	• 1.02<br>• 0.95<br>• 0.82	• 0.38<br>• 0.41<br>• 0.45	• 2.75<br>• 2.19<br>• 1.52	• 1.00<br>• 1.00<br>• 0.64	• 0.96<br>• 0.91<br>• 0.53
Sexual desire	• Victims: 31/37<br>• Non-victims: 69/33<br>• Total: 100/70	• 2.07<br>• 0.98<br>• 1.10	• 0.75<br>• 0.42<br>• 0.59	• 5.70<br>• 2.28<br>• 2.03	• 0.21<br>• 1.00<br>• 0.87	• 0.15<br>• 0.97<br>• 0.75

Meaningful friendships	• Victims: 52/16<br>• Non-victims: 89/13<br>• Total: 141/29	• 0.45<br>• 0.83<br>• 0.55	• 0.12<br>• 0.25<br>• 0.24	• 1.60<br>• 2.67<br>• 1.26	• 0.25<br>• 0.77<br>• 0.22	• 0.21<br>• 0.75<br>• 0.15
Social support	• Victims: 53/15<br>• Non-victims: 91/11<br>• Total: 144/26	• 5.37<br>• 1.16<br>• 2.58	• 1.10<br>• 0.33<br>• 1.05	• 26.21<br>• 4.09<br>• 6.33	• 0.03<br>• 1.00<br>• 0.05	• 0.03<br>• 0.81<br>• 0.03
Impediment in carrying out normal daily activities due to pain	• Victims: 29/39<br>• Non-victims: 18/84<br>• Total: 47/123	• 12.5<br>• 10.0<br>• 12.3	• 3.21<br>• 2.67<br>• 4.83	• 48.3<br>• 37.4<br>• 31.2	<.001<br><.001<br><.001	• <.001<br>• <.001<br>• <.001
Regular physical activity/sport	• Victims: 18/50<br>• Non-victims: 42/60<br>• Total: 60/110	• 0.37<br>• 1.05<br>• 0.66	• 0.12<br>• 0.47<br>• 0.35	• 1.13<br>• 2.33<br>• 1.25	• 0.09<br>• 1.00<br>• 0.26	• 0.08<br>• 0.90<br>• 0.20
Tiredness/absence of energy even in the early morning	• Victims: 47/21<br>• Non-victims: 40/62<br>• Total: 87/83	• 5.83<br>• 4.92<br>• 5.77	• 1.90<br>• 2.09<br>• 2.98	• 17.9<br>• 11.6<br>• 11.2	• 0.002<br>• <.001<br>• <.001	• 0.002<br>• <.001<br>• <.001
High emotionality (very intense expression and experience of emotional states)	• Victims: 55/13<br>• Non-victims: 59743<br>• Total: 114/56	• 2.21<br>• 1.68<br>• 2.13	• 0.65<br>• 0.75<br>• 1.10	• 7.52<br>• 3.79<br>• 4.11	0.22<br>0.22<br>0.03	• 0.20<br>• 0.20<br>• 0.02
More than 4 sexual partners during the course of life	• Victims: 42/26<br>• Non-victims: 39/63<br>• Total: 81/89	• 2.92<br>• 1.54<br>• 2.25	• 1.05<br>• 0.68<br>• 1.21	• 8.10<br>• 3.46<br>• 4.17	• 0.04<br>• 0.31<br>• 0.01	• 0.04<br>• 0.29<br>• 0.01
Age of first sexual intercourse less than 14 years	• Victims: 5/63<br>• Non-victims: 5/97<br>• Total: 10/160	• 2.63<br>• 5.95<br>• 4.31	• 0.27<br>• 0.64<br>• 0.88	• 24.9<br>• 55.2<br>• 20.9	• 0.64<br>• 0.15<br>• 0.09	• 0.39<br>• 0.11<br>• 0.07

Pain during sexual intercourse	• Victims: 26/42<br>• Non-victims: 13/89<br>• Total: 39/131	• 1.68<br>• 0.83<br>• 1.60	• 0.60<br>• 0.25<br>• 0.77	• 4.74<br>• 2.77<br>• 3.30	• 0.44<br>• 1.00<br>• 0.27	• 0.32<br>• 0.77<br>• 0.20

* Haldane-Anscombe correction
